# Leptin Induces Mitosis and Activates the Canonical Wnt/β-Catenin Signaling Pathway in Neurogenic Regions of *Xenopus* Tadpole Brain

**DOI:** 10.3389/fendo.2017.00099

**Published:** 2017-05-08

**Authors:** Melissa Cui Bender, Christopher J. Sifuentes, Robert J. Denver

**Affiliations:** ^1^Department of Molecular, Cellular and Developmental Biology, University of Michigan, Ann Arbor, MI, USA

**Keywords:** leptin, neurogenesis, *Xenopus*, metamorphosis, Wnt/β-catenin

## Abstract

In addition to its well-known role as an adipostat in adult mammals, leptin has diverse physiological and developmental actions in vertebrates. Leptin has been shown to promote development of hypothalamic circuits and to induce mitosis in different brain areas of mammals. We investigated the ontogeny of leptin mRNA, leptin actions on cell proliferation in the brain, and gene expression in the preoptic area/hypothalamus of tadpoles of *Xenopus laevis*. The level of leptin mRNA was low in premetamorphic tadpoles, but increased strongly at the beginning of metamorphosis and peaked at metamorphic climax. This increase in leptin mRNA at the onset of metamorphosis correlated with increased cell proliferation in the neurogenic zones of tadpole brain. We found that intracerebroventricular (i.c.v.) injection of recombinant *Xenopus* leptin (rxLeptin) in premetamorphic tadpoles strongly increased cell proliferation in neurogenic zones throughout the tadpole brain. We conducted gene expression profiling of genes induced at 2 h following i.c.v. injection of rxLeptin. This analysis identified 2,322 genes induced and 1,493 genes repressed by rxLeptin. The most enriched Kyoto Encyclopedia of Genes and Genomes term was the canonical Wnt/β-catenin pathway. Using electroporation-mediated gene transfer into tadpole brain of a reporter vector responsive to the canonical Wnt/β-catenin signaling pathway, we found that i.c.v. rxLeptin injection activated Wnt/β-catenin-dependent transcriptional activity. Our findings show that leptin acts on the premetamorphic tadpole brain to induce cell proliferation, possibly acting *via* the Wnt/β-catenin signaling pathway.

## Introduction

Leptin is a protein hormone secreted by adipocytes that signals energy stores to the adult brain. The plasma concentration of leptin fluctuates in proportion to fat mass, and leptin acts on feeding control centers in the hypothalamus to suppress food intake and increase whole body metabolism (1). Leptin (*lep*) and its receptor (LepR) are widely expressed, and the hormone has been shown to have pleiotropic actions in physiology and development of diverse vertebrate species (2, 3). The actions of leptin are mediated by the long form of the LepR (LepRb), which engages several intracellular signaling pathways, especially the Janus kinase 2/signal transducer and activator of transcription (JAK2/STAT) (4, 5) and phosphoinositide 3-kinase (PI3K) signaling pathways (6, 7).

Recent findings support that leptin has important roles in neurological development, especially development of leptin-responsive feeding control circuits in the hypothalamus. Leptin-deficient mice (*ob/ob*) have reduced brain weight, volume, and DNA content that can be restored by injecting leptin (4, 8). Leptin induces mitosis in several brain areas in rodents (8–10), and in tissue culture (11, 12), and this role for leptin is likely related to the brain size defects seen in *ob/ob* mice. The hormone also promotes formation of neuronal projections among hypothalamic nuclei involved with feeding (13–16).

In rodents, serum leptin concentration increases markedly in the neonate, then declines in the juvenile adult (17–19). This “postnatal leptin surge” may play a critical role in the development of the hypothalamic feeding control circuit (13, 15, 16). Cells in the ventricular zone (VZ)/subventricular zone (SVZ) of the third ventricle (3V) of neonates express functional LepR; LepR mRNA expression declines in the VZ/SVZ through development then appears in the arcuate nucleus and ventromedial hypothalamus (18). The LepR is expressed within the VZ of the 3V in embryonic/fetal brain (18, 20) and these neural progenitor/stem cells (NSCs) may be precursors of hypothalamic feeding control and hypophysiotropic neurons of the adult (21).

Genes for *lep* have now been isolated from numerous mammalian species, birds, reptiles, amphibians, and fishes (2, 3, 22). Our earlier findings in *Xenopus* support that the adipostat function of leptin was present in the earliest tetrapods (23, 24). By contrast, a role for leptin in feeding and energy balance in fishes remains unresolved (2, 3). Like mammals, tadpoles of *Xenopus laevis* develop competence to respond to leptin signaling during the postembryonic developmental period of metamorphosis (Melissa Cui Bender and Robert J. Denver, unpublished data). We found that functional LepR is expressed in regions surrounding the 3V of premetamorphic tadpole brain, suggesting that leptin can act within tadpole neurogenic zones. In the current study, we investigated whether leptin can promote mitosis in developing *Xenopus* tadpole brain by administering recombinant *Xenopus* leptin (rxLeptin) to premetamorphic tadpoles by intracerebroventricular (i.c.v.) injection, then we analyzed cells in M phase of the cell cycle using immunohistochemistry (IHC) for phosphorylated histone 3 (pH3). We also conducted a gene expression screen for early (2 h after i.c.v. rxLeptin injection) leptin-induced transcriptional changes in tadpole preoptic area/hypothalamus. This screen identified the canonical Wnt/β-catenin signaling pathway as the major intracellular signaling pathway induced by leptin in premetamorphic tadpole brain. Using electroporation-mediated (EM) gene transfer of a Wnt/β-catenin-responsive reporter plasmid into tadpole brain, we provide additional evidence that leptin activates functional Wnt/β-catenin signaling.

## Materials and Methods

### Animal Care and Use

We obtained *X. laevis* tadpoles from in-house breeding and raised them in dechlorinated tap water maintained at 21–23°C with a 12L:12D photoperiod. Tadpoles were fed frog brittle twice daily (NASCO, Fort Atkinson, WI, USA) and developmental stages were determined using the Nieuwkoop–Faber (NF) staging table (25). We anesthetized NF stage 50 *X. laevis* tadpoles (premetamorphic tadpoles) in a buffered solution of 0.002% benzocaine (Sigma) before administering i.c.v. injection of rxLeptin [produced as described by Crespi and Denver (23)], or plasmid injections for EM gene transfer (described below). For i.c.v. injection, we used a Drummond microinjector to deliver 50–150 nL of solution containing 0.6% saline, rxLeptin (20 ng/g BW) or plasmid DNA, plus 0.01% fast green dye to the area of the 3V as described previously (23, 24, 26). We chose this dose of rxLeptin based on our previously published work that showed that i.c.v. injection caused a dose-dependent suppression of food intake in the Western spadefoot toad, with 20 ng/g BW rxLeptin causing maximal suppression (23). Animals were killed by immersion in 0.1% benzocaine for 2 min before tissue harvest. All procedures involving animals were conducted under an approved animal use protocol (PRO00006809) in accordance with the guidelines of the Institutional Animal Care and Use Committee at the University of Michigan.

### RNA Isolation for Gene Expression Analyses

For developmental analysis of *lep* mRNA, we extracted total RNA from whole animals beginning at NF stage 45. For NF stages 45–54, we pooled three animals per replicate and for NF stages 58–66, one animal per replicate (*n* = 5–6/NF stage). We also dissected and isolated total RNA from the portion of the carcass containing the fat pads (we removed tail and other organs; we refer to this as “adipose tissue”), liver, brain, and gut from tadpoles throughout metamorphosis (NF stages 50, 54, 58, 62, 66) for analysis of *lep* mRNA (*n* = 6/NF stage). For analysis of gene expression by microarray and reverse transcriptase quantitative real-time PCR (RTqPCR), we injected tadpoles i.c.v. with 0.6% saline or rxLeptin (20 ng/g BW), and 2 h later, we killed the animals, removed the brain, and microdissected the middle region of the brain (*n* = 6/treatment; the region of the diencephalon containing the preoptic area and hypothalamus, the neuroendocrine and feeding control regions of the brain) (27). We isolated RNA using the Trizol reagent (Invitrogen) following the manufacturer’s instructions.

For RTqPCR analysis of gene expression, we developed SYBR Green assays that spanned exon–exon boundaries for each gene. We treated 1 µg total RNA with 1.5 U RNase-free DNase I (Roche, Indianapolis, IN, USA) to digest contaminating genomic DNA, then reverse-transcribed the RNA into cDNA using 250 ng random hexamers and Superscript II Reverse Transcriptase (Invitrogen) following the manufacturer’s instructions [see also Ref. (28, 29)]. Minus RT controls were included. Oligonucleotides used for RTqPCR are given in Table 1. We conducted quantitative, real-time PCR using the ABsolute™ Blue qPCR SYBR Green Low Rox Mix (ABgene), and reactions were run on an ABI 7500 Fast qPCR machine. Relative quantities were determined using standard curves generated with pooled cDNAs.

**Table 1:** **Sequences of oligonucleotide used to validate microarray results by SYBR Green reverse transcriptase quantitative real-time PCR**.

Gene name	Forward	Reverse
*ctnnb1*	5′ GAATTGGCCACTCGAGCAA 3′	5′ ACCTGGTCCTCGTCATTAAGC 3′
*sox8*	5′ GGGCAAACTGTGGCGTTTA 3′	5′ CTCAGCCTCCTCCACAAAGG 3′
*socs3*	5′ AGAACCTACGCATCCAGTGTGA 3′	5′ GGCACTTCGTGGGTCAGTCT 3′
*arrb2*	5′ TCAGTCAGACAATACGCAGACATC 3′	5′ GCCACCGGGCCACTTGTAC 3′
*dab2*	5′ CAGCAGCTGCCACTGGAA 3′	5′ ATTGTTGTGCGTGAGAGTTTAC 3′
*mad2l1*	5′ AAGAACTTGCAACCGTTAAACT 3′	5′ TCACGAACAATGCCGTCTTTC 3′
*rpL8*	5′ TTTGCTGAAAGAAATGGCTACATC 3′	5′ CACGGCCTGGATCATGGA 3′

### IHC for pH3 and Phosphorylated STAT3 (pSTAT3)

We analyzed the effects of i.c.v. injection of rxLeptin on mitosis in the premetamorphic tadpole brain using IHC for pH3. The serine 10 at the amino terminus of H3 is phosphorylated during late G2 through M phase of the cell cycle and is therefore used to detect dividing cells [for *X. laevis* brain: (30–32)]. We administered two i.c.v. injections of 0.6% saline or rxLeptin (200 ng/g BW) (23) into the region of the 3V of NF stage 50 tadpoles; the second injection was 24 h after the first, and tadpoles were killed 48 h after the first injection (i.e., 24 h after the second injection). We then processed brains for IHC for pH3 following the method of Denver et al. (30). Briefly, we prepared 10 µm thick transverse cryosections through entire tadpole brains and immunostained sections with a rabbit antiserum against human pH3 (1:500; Cat. #0650 EMD Millipore, Billerica, MA, USA). Primary immune complexes were detected using a secondary antibody conjugated with Cy3 (1:500, Jackson ImmunoResearch Laboratories, Inc., West Grove, PA, USA). For IHC for pSTAT3, a marker for activated LepR, we administered i.c.v. injections of 0.6% saline or rxLeptin (20 ng/g BW) (24) to NF stage 50 tadpoles and collected brains 1 h later for fixation and sectioning. We conducted IHC for pSTAT3 on *Xenopus* brain as described previously (24) using an antiserum generated against a phosphopeptide AP[pY]LK from mouse STAT3 (Tyr705; 1:200; Cat. # 9131, Cell Signaling), which is 100% conserved with *X. laevis* STAT3 (24, 33). Images of immunostained slides were captured using an Olympus XI-81 microscope, and the total number of pH3 positive cells per brain was counted. Five brains were analyzed per treatment. *Xenopus* neuroanatomy is based on Tuinhof et al. (34) with modifications by Yao et al. (35).

### DNA Microarray Analysis of Gene Expression after i.c.v. rxLeptin Injection

We conducted DNA microarray analysis on total RNA isolated from the preoptic area/hypothalamus of NF stage 54 *X. laevis* tadpoles (~0.2 g BW) 2 h after i.c.v. injection of 0.6% saline or rxLeptin (20 ng/g BW). We isolated total RNA from microdissected brain regions of three replicate pools (three brains per pool) per treatment. Samples were hybridized to the Affymetrix GeneChip *Xenopus laevis* Genome 2.0 Array (*n* = 3/treatment) at the Affymetrix and Microarray Core Facility at the University of Michigan. We selected a subset of the induced genes to cover a range of expression values (fold change) for validation by RTqPCR (Table 1).

#### Microarray Data Background Correction, Normalization, and Expression Quantification

We used the Robust Multichip Average method (36) from the Affy package (v1.52.0) (37) to calculate background-corrected, normalized expression values. Genefilter (v1.56.0) (38) was used to remove uninformative probesets (internal control probesets, or probesets with low variance or background level expression values).

#### Microarray Data Differential Expression Analysis and Annotation

Principal component analysis plots of the normalized, filtered samples showed samples of the same treatment (saline or rxLeptin) clustered together, and that the two treatments clustered separately. We used the limma package (v3.30.2) (39) to apply empirically derived array weights to individual samples, which were then used to calculate differential expression values (40). Annotations for each gene were added using the annotation file supplied by Affymetrix for the *X. laevis* Genome 2.0 Array.

#### Gene Ontology (GO) and Pathway Analysis

Gene ontology term enrichment analysis was conducted using a log_2_ fold change (log_2_FC) ranked list from limma, containing genes with a Benjamini and Hochberg (BH)-corrected *p*-value, or false-discovery rate (FDR) <0.05, as input into clusterProfiler (v3.2.4) (41). This analysis determines which molecular function, biological process, or cellular component GO terms are positively or negatively enriched in rxLeptin-treated samples compared with saline-treated controls, at a *p*-value < 0.05, while taking into account the magnitude and direction of change. Due to the hierarchical and redundant nature of GO terms, we obtained a summarized list of GO terms using Revigo to remove redundant terms (42).

We conducted Kyoto Encyclopedia of Genes and Genomes (KEGG) (43) pathways enrichment analysis using a log_2_FC ranked list of all differential gene expression data from limma (FDR < 0.05) as input into clusterProfiler (v3.2.4) (41). An additional KEGG module (highly annotated functional units of a metabolic network) enrichment analysis was conducted using the same log_2_FC ranked list as input into clusterProfiler (v3.2.4). Gene expression values for KEGG pathways were plotted on the respective pathways using the pathview package (v1.14.0) (44).

Enriched biological processes GO terms, KEGG pathways, and KEGG modules were displayed using the Enrichment Map application (v2.2.0) (45) in Cytoscape (v3.4.0) (46).

### EM Gene Transfer and *In Vivo* Reporter Assay

We used EM gene transfer (30, 47–50) and i.c.v. microinjection of 0.6% saline or rxLeptin (20 ng/g BW) to investigate whether leptin can activate the Wnt/β-catenin signaling pathway in tadpole brain *in vivo*. We divided NF stage 50 tadpoles into three groups for bipolar electroporation. Tadpoles were electroporated with a DNA mixture containing pRenilla-null (50 ng/µL; for dual-luciferase assay normalization), pCMV-eGFP (500 ng/µL; to visualize transfection efficiency), and one of the following firefly luciferase reporter vectors (each at 1 μg/μL): pGL4.23 empty (negative control), GAS-luciferase (positive control for leptin activity) (23, 24) or pGL4.23-6TCF (reporter of activated canonical Wnt/β-catenin signaling). The pGL4.23-6TCF vector contains six T cell factor (TCF) sites upstream of a minimal promoter driving luciferase expression, and therefore reports activation of the canonical Wnt/β-catenin signaling pathway (gift of Dr. Ken Cadigan). A separate group of tadpoles received pGL4.23-6TCF, pRenilla-null, and pCMV-EGFP plus a constitutively active β-catenin expression vector pcDNA3-S33Y β-catenin (51) (gift of Dr. Eric Fearon) or an empty vector (pCMVneo-empty). Plasmid concentrations for electroporation are based on Yao et al. (52). Twenty-four hours after EM gene transfer we screened tadpoles for strong GFP expression, and then separated them into eight groups: pGL4.23-empty reporter, saline, or rxLeptin; GAS-luciferase, saline, or rxLeptin; pGL4.23-6TCF, saline, or rxLeptin; pGL4.23-6TCF, pCMVneo-empty, or pcDNA3-S33Y β-catenin. Tadpoles receiving i.c.v. injections were given saline or rxLeptin (20 ng/g BW). Two hours after injection, tadpoles were killed and brains harvested for dual-luciferase assay following the protocol described by Yao et al. (52). We had a sample size of eight tadpoles per treatment.

### Data and Statistical Analysis

We analyzed data for gene expression by RTqPCR, pH3 cell counts and dual-luciferase assay data by one-way ANOVA or by unpaired Student’s *t*-test (*p* < 0.05). Derived values were log_10_-transformed before statistical analysis. Fisher’s least squares difference *post hoc* test was used to separate the means following ANOVA. We used the SYSTAT 13.0 computer program (SPSS Inc.).

## Results

### Ontogeny of *lep* mRNA in Tadpoles during Metamorphosis, and Effects of rxLeptin on Mitosis in Premetamorphic Tadpole Brain

**Figure 1:**
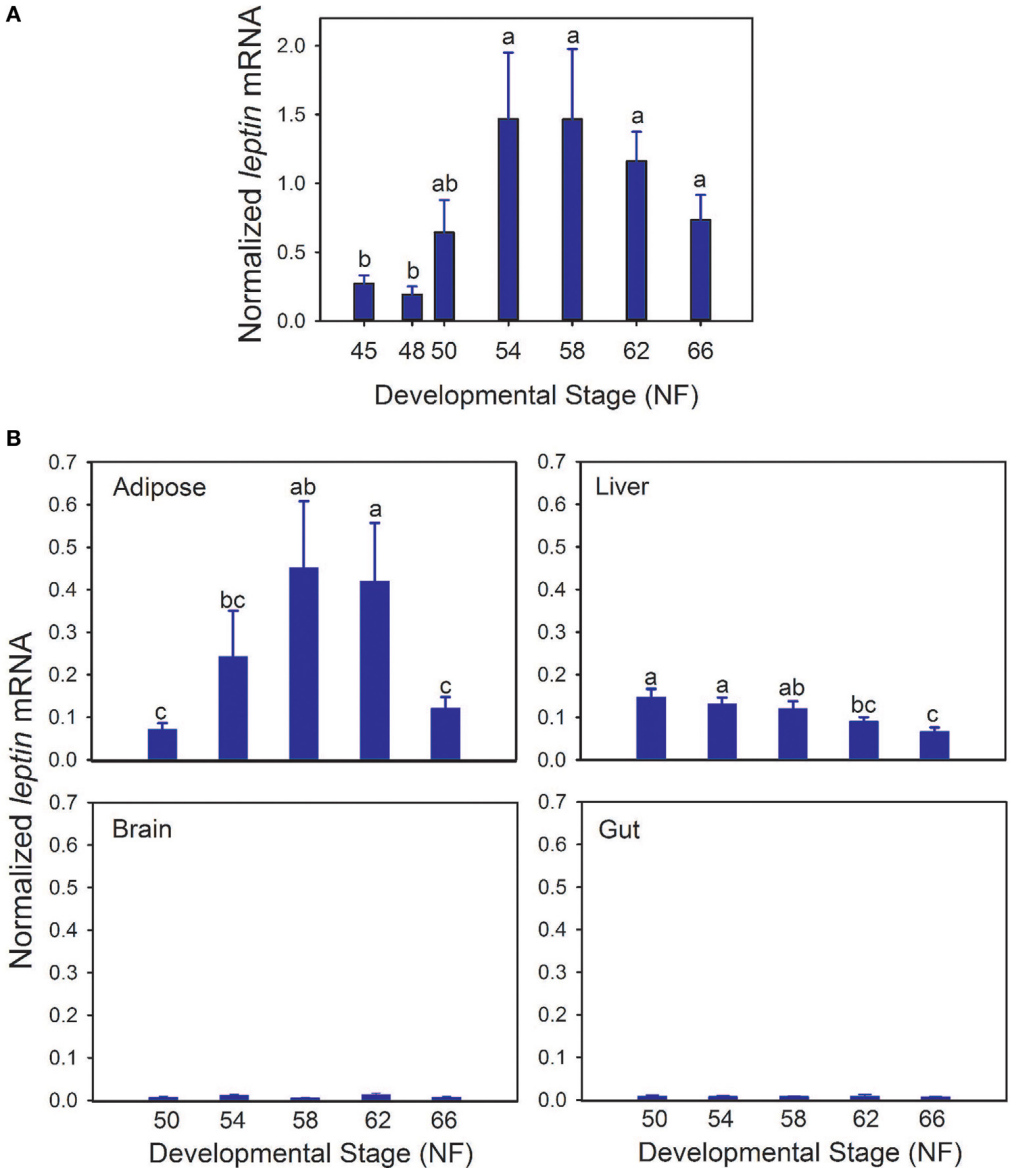
**Transcription of the *lep* gene is activated during tadpole metamorphosis**. **(A)** Whole body *lep* mRNA increases during tadpole metamorphosis. **(B)** Developmental changes in *lep* mRNA in four tadpole tissues during metamorphosis. We analyzed *lep* mRNA by reverse transcriptase quantitative real-time PCR. Note that the scales of the graphs in panel **(B)** are not directly comparable to the graph in panel **(A)** since the samples were analyzed in separate assays using a relative quantification method (see Materials and Methods). Means with the same letter are not significantly different [*p* < 0.05; Fisher’s least squares difference test; *n* = 5–6/Nieuwkoop–Faber (NF) stage].

**Figure 2:**
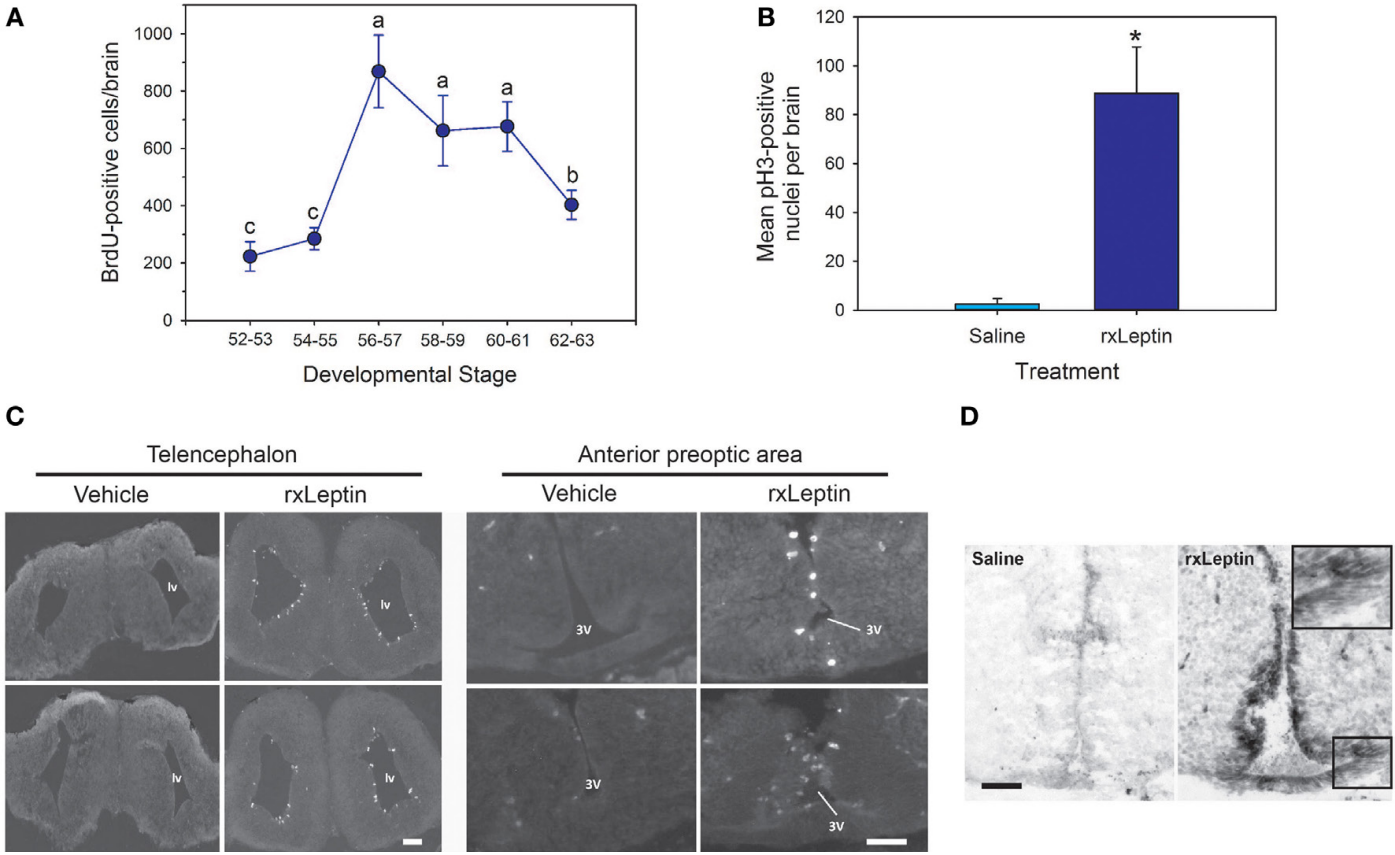
**Leptin induces mitosis in premetamorphic tadpole brain**. **(A)** Changes in cell proliferation in tadpole brain throughout metamorphosis analyzed by BrdU incorporation [data are modified from Denver et al. (30); reproduced with permission]. **(B)** Quantification of pH3 positive cells in tadpole brain following intracerebroventricular injection of saline or recombinant *Xenopus* leptin (rxLeptin) (200 ng/g BW). Tadpoles were given two injections, the second 24 h after the first, and then they were killed 48 h after the first injection. *Denotes a statistically significant difference (*p* < 0.05; Student’s unpaired t-test). **(C)** Images of transverse sections of the region of the telencephalon [lateral ventricle (lv)] and anterior preoptic area [location of neurosecretory neuron cell bodies; third ventricle (3V)] of tadpole brain stained for pH3. Scale bars = 120 µM. **(D)** Induction of pSTAT3 immunoreactivity in cells located in the ventricular zone (VZ)/subventricular zone (SVZ) of premetamorphic (Nieuwkoop–Faber stage 50) tadpole brain by rxLeptin (20 ng/g BW; 1 h). The inset shows a higher magnification view of the VZ/SVZ with elongated cells undergoing migration out of the neurogenic zone.

Whole body *lep* mRNA increased 3.3-fold between stages 48 and 50, which is immediately before the onset of metamorphosis (*p* < 0.001, ANOVA; Figure 1A). The mRNA level increased further (7.6-fold) from stages 50 to 54, and remained elevated throughout metamorphosis and in the postmetamorphic frog (NF stage 66). The pattern of changes in *lep* mRNA in adipose tissue during metamorphosis was similar to whole body (Figure 1B; *p* = 0.002, ANOVA). By contrast, *lep* mRNA decreased in liver (*p* = 0.001) and was low and unchanged in brain throughout metamorphosis (Figure 1B). These findings, and other results from our laboratory support that fat tissue is the major source of *lep* mRNA in the tadpole (Melissa Cui Bender and Robert J. Denver, unpublished data). This developmental pattern of *lep* mRNA paralleled changes in cell proliferation in tadpole brain during metamorphosis (Figure 2A) (30). Injection of rxLeptin strongly increased pH3-immunoreactivity (35-fold; *p* < 0.0001; Student’s unpaired *t*-test) in cells within the VZ/SVZ throughout the tadpole brain (Figures 2B,C). Also, i.c.v. rxLeptin injection (1 h) induced the appearance of pSTAT3 immunoreactivity (pSTAT3-ir) in the VZ/SVZ in the region of the 3V (Figure 2D), supporting the expression of functional LepR in these cells. The inset in Figure 2D shows a high magnification view of the VZ/SVZ with elongated cells undergoing migration out of the neurogenic zone.

### i.c.v. rxLeptin Injection Induces Rapid Transcriptional Responses in Early Premetamorphic (NF Stage 54) Tadpole Brain

**Figure 3:**
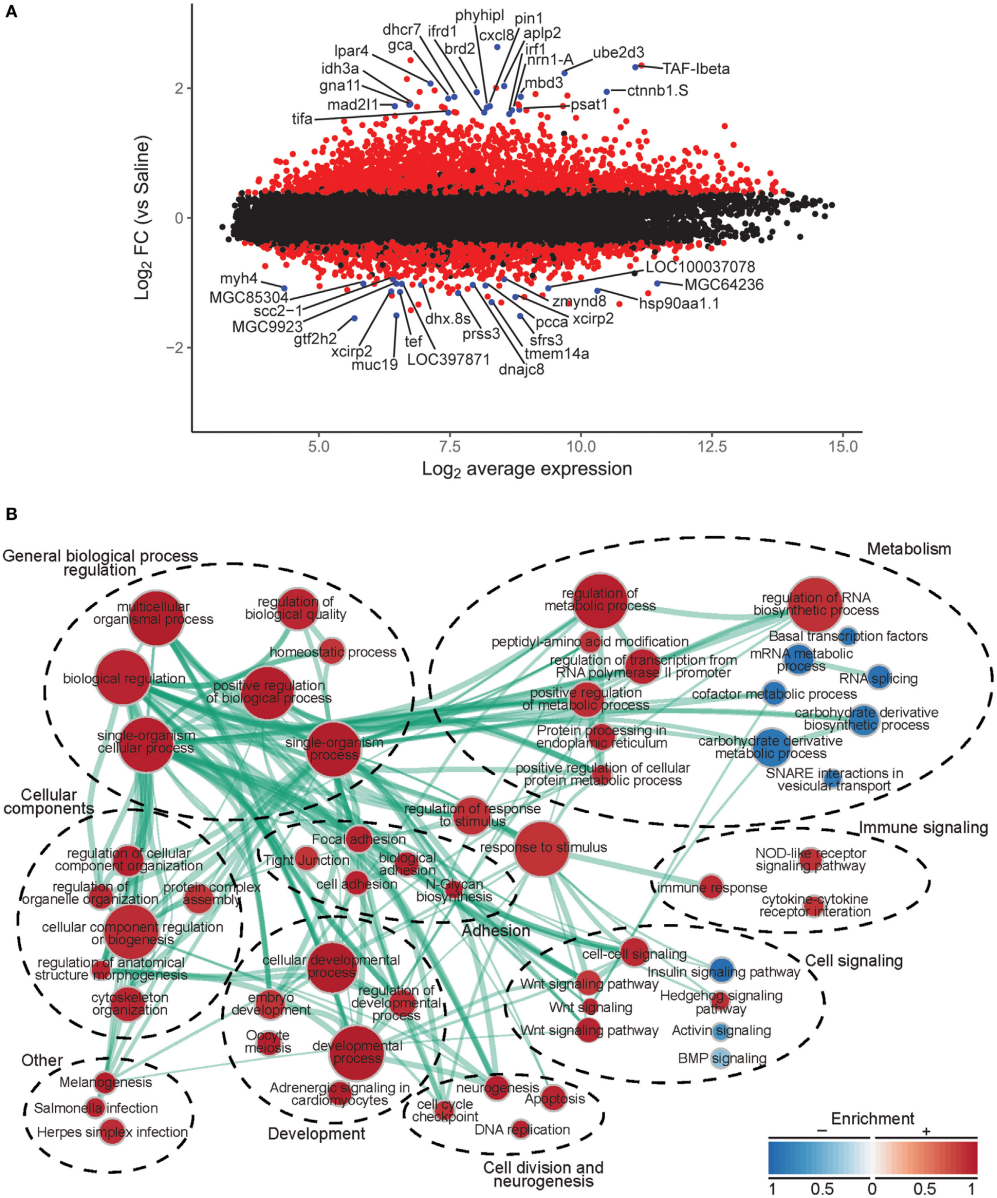
**Analysis of microarray data from preoptic area/hypothalamus of saline or recombinant *Xenopus* leptin (rxLeptin)-injected [intracerebroventricular (i.c.v.) 20 ng/g BW; 2 h] Nieuwkoop–Faber stage 54 tadpoles**. **(A)** Transformed log ratio and mean average plot [log_2_ fold change (log_2_FC) vs. average expression] of saline vs. rxLeptin-injected tadpole mRNA levels (see Materials and Methods). Dots represent genes: red = genes with a false-discovery rate <0.05; blue = top 20 differentially expressed and annotated *Xenopus laevis* genes; black = all other genes. **(B)** Biological processes, pathways, and modules affected by i.c.v. rxLeptin injection. Enrichment map of enriched biological process gene ontology terms (redundancy reduced, *p* < 0.05), Kyoto Encyclopedia of Genes and Genomes (KEGG) pathways (*p* < 0.05), and KEGG modules. Circular node color reflects positive (red) or negative (blue) enrichment. Node color intensity reflects the degree of enrichment relative to the most highly enriched in either direction. Lines (green) represent significant genetic information shared between connected nodes (above 50%), and line thickness represents the degree of shared information.

To investigate the molecular basis for leptin action on tadpole brain, we conducted microarray analysis on the region of the diencephalon containing the preoptic area/hypothalamus of NF stage 54 tadpoles at 2 h following i.c.v. injection of 0.6% saline or rxLeptin injection (20 ng/g BW). The transformed log ratio (M) and mean average (A) plot (MA plot) in Figure 3A shows that the data are normally distributed, with a zero-centered transformed log ratio. Additionally, this plot shows that genes with a significant BH-adjusted *p*-value (FDR < 0.05), plotted in red, are distributed evenly across MA expression levels, indicating an ability to detect differentially expressed genes at any range of expression. This plot also indicates that an absolute log2 fold change (log2FC) as low as ~0.5 can be detected with confidence (FDR < 0.05). This analysis identified 2,322 induced and 1,493 repressed genes (Table S1 in Supplementary Material). The top 20 induced and repressed genes are given in Tables 2 and 3, respectively. The microarray dataset has been deposited in the Gene Expression Omnibus archive at the National Center for Biotechnology Information (GEO accession #GSE97243).

**Table 2:** **Top twenty genes with annotation induced by recombinant *Xenopus* leptin in premetamorphic tadpole brain**.

Gene symbol	Gene title	Entrez gene ID	log_2_ fold change	Adjusted *p*-value
*LOC100036815/cxcl8*	Hypothetical protein LOC100036815/interleukin-8	100036815	2.63	0.00001
*ctnnb1*	Beta-catenin protein	399274	2.32	0.00013
*ube2d3*	Ubiquitin-conjugating enzyme E2D 3 (UBC4/5 homolog)	403384	2.23	0.00018
*lpar4*	Lysophosphatidic acid receptor 4	779407	2.07	0.00018
*aplp2*	Amyloid beta (A4) precursor-like protein 2	431790	2.03	0.00018
*set/TAF-Ibeta1*	SET nuclear oncogene///TAF-Ibeta1	379599///399349	1.94	0.00014
*brd2*	RING3 protein	779057	1.94	0.00028
*mbd3*	Methyl-CpG binding domain protein 3	398135	1.87	0.00018
*dhcr7*	7-Dehydrocholesterol reductase	379273	1.87	0.00028
*gca*	Grancalcin, EF-hand calcium binding protein	444080	1.84	0.00014
*idh3a*	Isocitrate dehydrogenase 3 (NAD+) alpha	444419	1.75	0.00017
*gna11*	Guanine nucleotide binding protein (G protein), alpha 11 (Gq class)	380535///779103	1.74	0.00026
*pin1*	Peptidylprolyl *cis*/*trans* isomerase, NIMA-interacting 1	503670	1.72	0.00018
*mad2l1*	MAD2 mitotic arrest deficient-like 1	380433	1.72	0.00074
*phyhipl*	Phytanoyl-CoA 2-hydroxylase interacting protein-like	447238	1.70	0.00037
*psat1*	Phosphoserine aminotransferase 1	494700	1.67	0.00013
*nrn1-A*	Neuritin 1-A	373709	1.66	0.00025
*ifrd1/MGC69123*	Interferon-related developmental regulator 1	379667///494857	1.63	0.00019
*tifa*	TRAF-interacting protein with forkhead-associated domain	734184	1.62	0.00026
*irf1*	Interferon regulatory factor 1	398826	1.60	0.00007

**Table 3:** **Top twenty genes with annotation repressed by recombinant *Xenopus* leptin in premetamorphic tadpole brain**.

Gene symbol	Gene title	Entrez gene ID	log_2_ fold change	Adjusted *p*-value
*gtf2h2/MGC81060*	General transcription factor IIH subunit 2	443754	−1.55	0.00027
*sfrs3*	Splicing factor, arginine serine-rich 3	380152	−1.51	0.00018
*muc19*	Mucin 19, oligomeric	378670	−1.50	0.00018
*tmem14a*	Transmembrane protein 14A	444418	−1.30	0.00613
*xcirp2*	Cold-inducible RNA binding protein 2	379484	−1.22	0.00391
*prss3*	Protease, serine, 3	447093	−1.16	0.01023
*tef*	Thyrotrophic embryonic factor	379940	−1.14	0.00018
*hsp90aa1.1/MGC82579*	Heat shock protein 90 kDa alpha family class A member 1	444024	−1.12	0.00030
*myh4*	Similar to myosin, heavy polypeptide 4, skeletal muscle	399414	−1.09	0.00145
*LOC100037078*	Hypothetical protein LOC100037078	100037078	−1.09	0.01290
*pcca*	Propionyl Coenzyme A carboxylase, alpha polypeptide	734347	−1.04	0.00330
*dnajc8*	DnaJ (Hsp40) homolog, subfamily C, member 8	447089	−1.03	0.00224
*dhx.8S/MGC80994*	DEAH-box helicase 8 S homolog	444315	−1.03	0.00145
*LOC397871*	Larval beta II globin	397871	−1.02	0.00106
*MGC85304*	MGC85304 protein	447105	−1.02	0.00207
*MGC99235*	MGC99235 protein	447690	−1.01	0.00053
*MGC64236*	Hypothetical protein MGC64236	379516	−1.01	0.00292
*scc2-1*	Scc2-1B	445865	−0.95	0.01224
*zmynd8*	Zinc finger, MYND-type containing 8	733267	−0.94	0.01584
*hmgn1/MGC64236*	High mobility group nucleosome binding domain 1	379516	−0.94	0.00244

**Figure 4:**
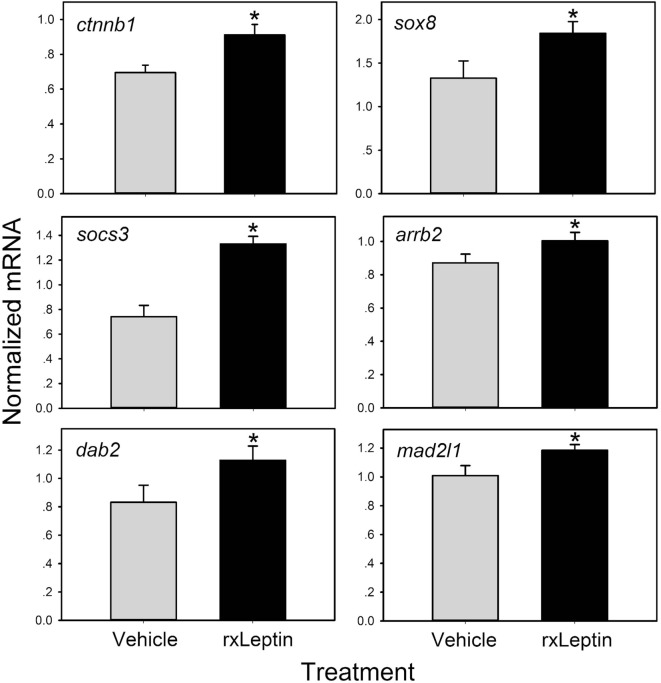
**Validation of leptin-induced genes in Nieuwkoop–Faber stage 54 *Xenopus laevis* tadpole preoptic area/hypothalamus by reverse transcriptase quantitative real-time PCR (RTqPCR)**. Tadpoles received an injection intracerebroventricular of 0.6% saline or recombinant *Xenopus* leptin (rxLeptin) (20 ng/g BW) and were killed 2 h later for tissue harvest for RNA isolation. Gene expression was analyzed by SYBR Green RTqPCR and normalized to the reference gene *rpL8* which was not affected by rxLeptin injection (data not shown). Asterisks indicate statistically significant differences from saline injected controls (*p < 0.05; unpaired Student’s *t*-test).

Using these differential expression data, we conducted several enrichment analyses, allowing us to classify the global gene regulation changes into several general categories (general biological process regulation, metabolism, cellular components, adhesion, immune signaling, cell signaling, development, cell division and neurogenesis, and others) and highlighting several biological processes or pathways of interest. The enrichment map in Figure 3B shows these data: enriched biological process GO terms (*p*-value < 0.15), KEGG pathways (*p*-value < 0.15), and KEGG modules. We validated a subset of the induced genes by RTqPCR (Figure 4).

**Figure 5:**
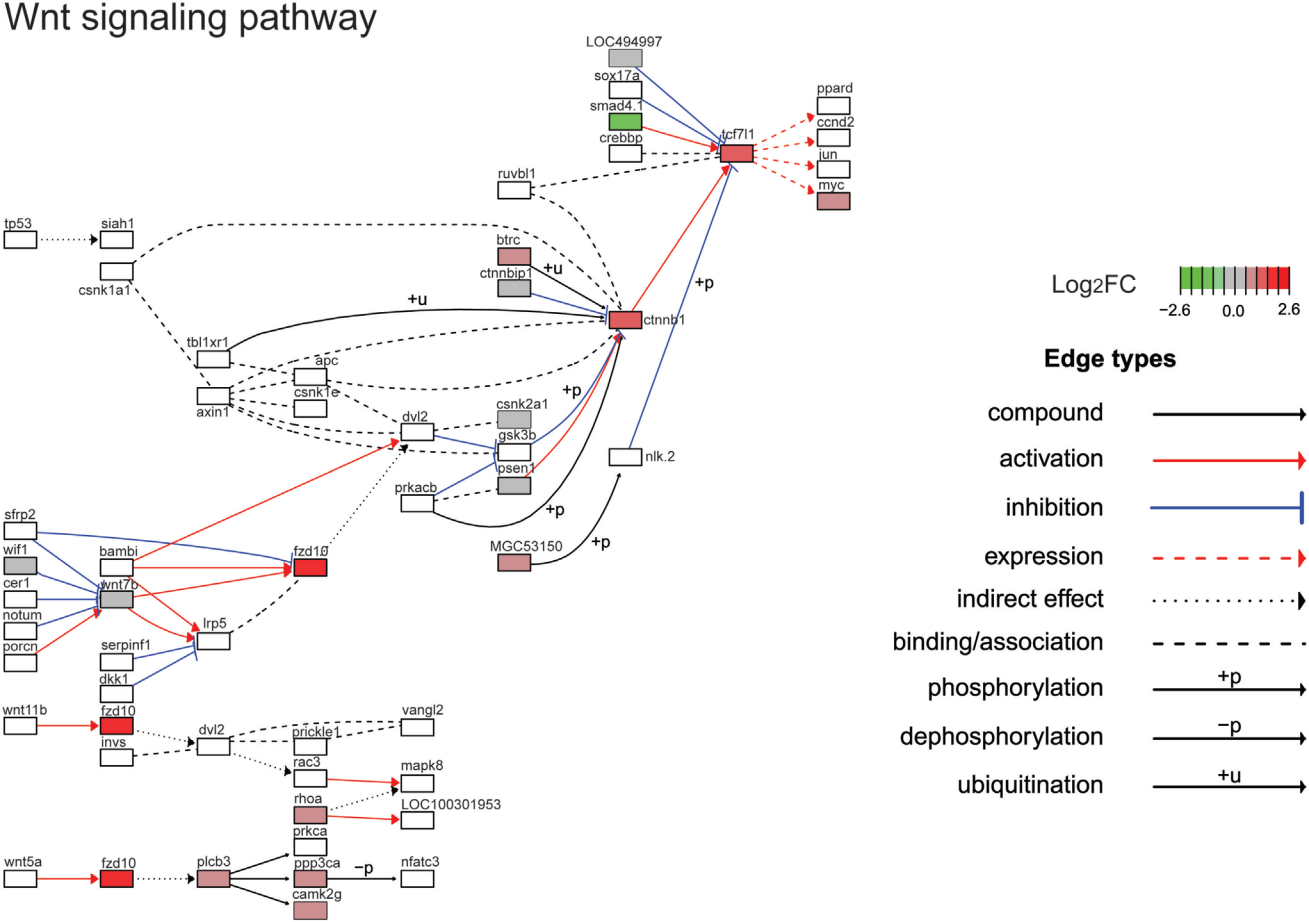
**Gene expression changes in the Wnt/β-catenin signaling pathway**. Shown is the Kyoto Encyclopedia of Genes and Genomes Wnt/β-catenin signaling pathway with differential gene expression values plotted for genes with false-discovery rate <0.05. The color of each gene represents the direction of regulation (red = positive; gray = no change; green = negative). log_2_FC = log_2_ fold change. The intensity of the color is the value relative to the most highly regulated gene (log_2_FC = 2.6) in the entire dataset.

**Figure 6:**
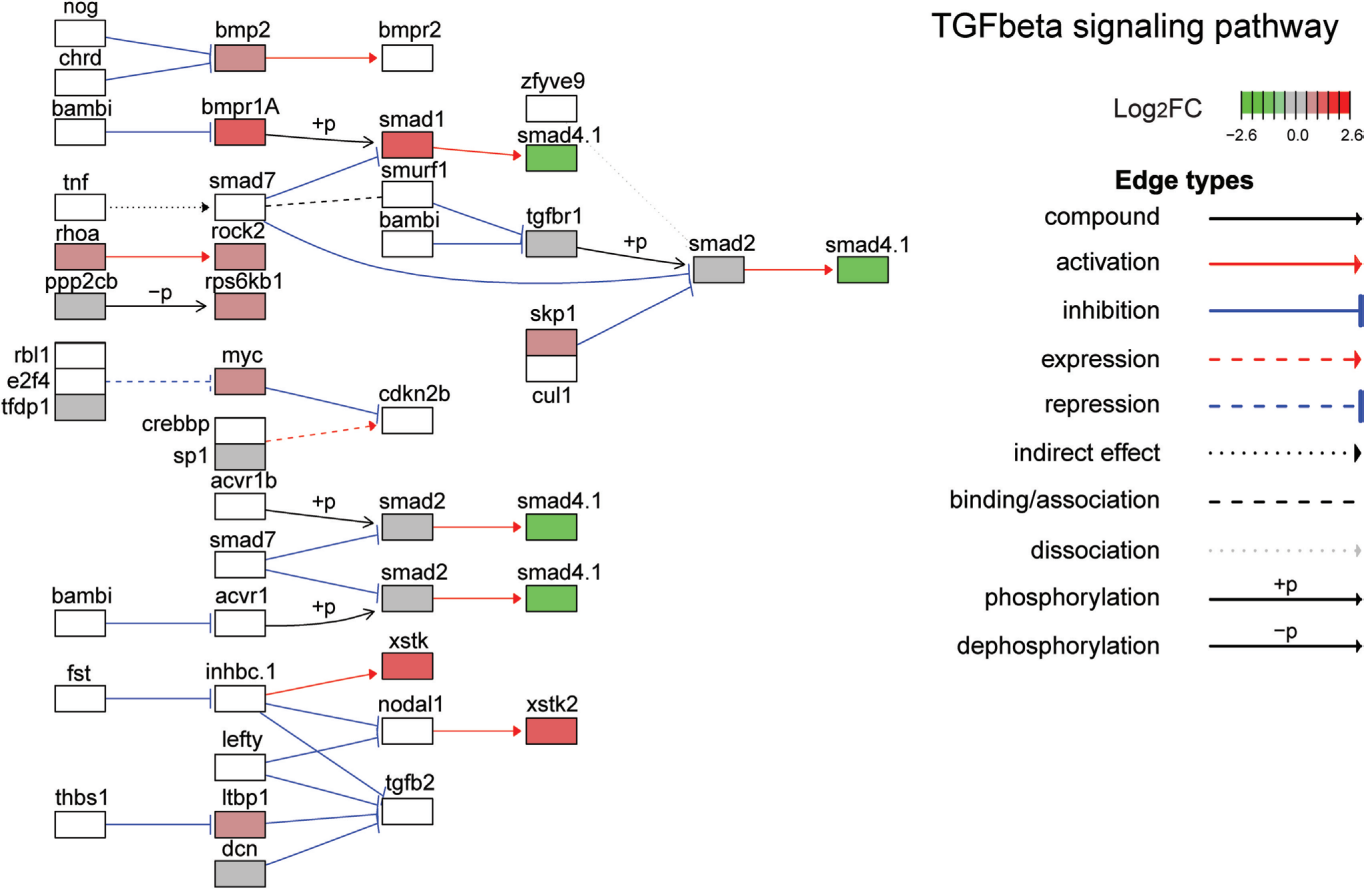
**Gene expression changes in the TGFβ signaling pathway**. Shown is the Kyoto Encyclopedia of Genes and Genomes TGFβ signaling pathway with differential gene expression values plotted for genes with false discover rate <0.05. The color of each gene represents the direction of regulation (red = positive; gray = no change; green = negative). log_2_FC = log_2_ fold change. The intensity of the color is the value relative to the most highly regulated gene (log_2_FC = 2.6) in the entire dataset.

The top ten GO terms involved developmental processes (Figure 3B; Table 4; Table S2 in Supplementary Material). Noteworthy is the Wnt/β-catenin signaling pathway, which was found by each enrichment analysis (biological process GO, KEGG pathway, KEGG module; Figure 5). Also, signaling by two TGFβ family members, bone morphogenetic protein (BMP) and activin, was among the enriched KEGG modules (Figure 6; Table S3 in Supplementary Material; tab KEGGmodules.filt). The hedgehog and insulin signaling pathways were also found to be enriched (Figures S1 and S2 in Supplementary Material).

**Table 4:** **Top ten gene ontology (GO) terms corresponding to developmental processes**.

ID	Description	Set size	Enrichment score	NES	*p*-Value	*p*.adjust	*q*-Values
GO:0044767	Single-organism developmental process	470	0.298665447	1.479435427	0.00101833	0.159221311	0.159221311
GO:0048856	Anatomical structure development	452	0.305758564	1.512476805	0.00101833	0.159221311	0.159221311
GO:0032502	Developmental process	473	0.305340626	1.513106572	0.001019368	0.159221311	0.159221311
GO:0044707	Single-multicellular organism process	433	0.301770432	1.490562662	0.001023541	0.159221311	0.159221311
GO:0032501	Multicellular organismal process	463	0.29984464	1.483930907	0.00102459	0.159221311	0.159221311
GO:0007275	Multicellular organism development	407	0.300744558	1.475210668	0.003089598	0.400102987	0.400102987
GO:0030154	Cell differentiation	203	0.319354548	1.47945519	0.004429679	0.491694352	0.491694352
GO:0009790	Embryo development	84	0.391329957	1.634661027	0.006157635	0.598060345	0.598060345
GO:0048513	Animal organ development	137	0.342563144	1.530163684	0.007900677	0.649743161	0.649743161
GO:0048869	Cellular developmental process	242	0.301733778	1.42896268	0.008583691	0.649743161	0.649743161

### Wnt/β-Catenin Signaling Is Activated in Premetamorphic Tadpole Brain by i.c.v. rxLeptin Injection

**Figure 7:**
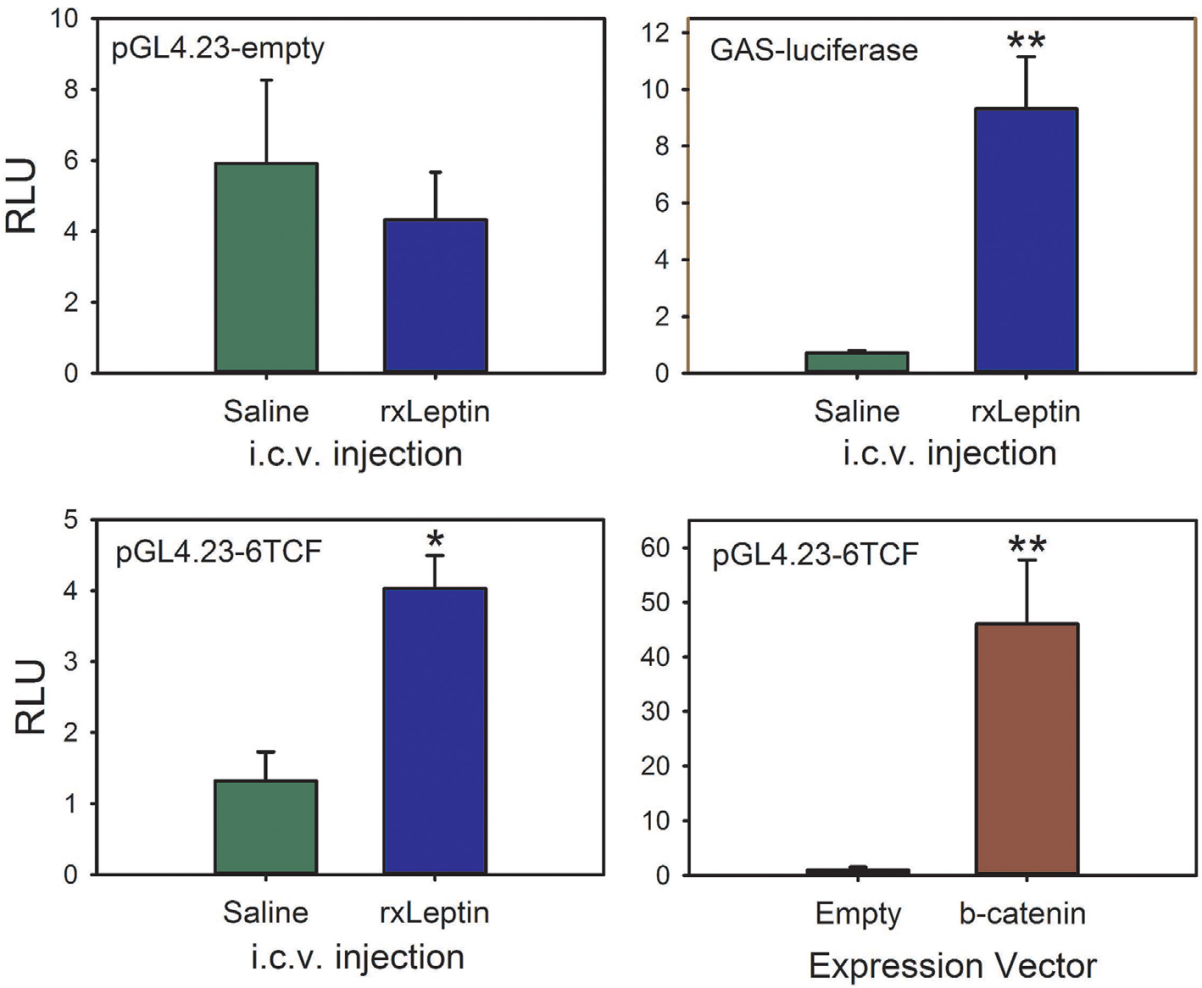
**The canonical Wnt/β-catenin signaling pathway is activated by leptin signaling in *Xenopus laevis* tadpole brain**. We injected plasmids into the region of the third ventricle of premetamorphic (Nieuwkoop–Faber stage 50) tadpoles and transfected them by biopolar electroporation-mediate gene transfer. Twenty-four hours after transfection, we screened tadpoles for EGFP expression, and then separated them into eight groups. The reporter vector is given at the top left of each panel. Tadpoles received intracerebroventricular (i.c.v.) injections of 0.6% saline or recombinant *Xenopus* leptin (rxLeptin) (20 ng/g BW); a separate group was cotransfected with the pGL4.23-6TCF reporter vector and either empty expression vector (pCMVneo-empty) or a vector that expresses constitutively active β-catenin (pcDNA3-S33Y β-catenin). Two hours after i.c.v. injection, tadpoles were killed and brains harvested for dual-luciferase assay. Asterisks indicate statistically significant differences (*p < 0.001, **p < 0.0001; *n* = 8 tadpoles/treatment).

To test if leptin can activate the canonical Wnt/β-catenin signaling pathway in tadpole brain *in vivo*, we conducted EM gene transfer with different reporter plasmids, and injected saline or rxLeptin i.c.v. Injection of rxLeptin did not alter luciferase activity in pGL4.23-empty vector-transfected brain, but caused a strong increase (12.9-fold; *p* < 0.0001, unpaired Student’s *t*-test) in luciferase driven by the GAS-luciferase vector, which reports pSTAT3 signaling induced by leptin binding to the LepR (Figure 7) (23, 24). This confirmed activation of LepR signaling in tadpole brain after i.c.v. rxLeptin injection. We observed a threefold increase in luciferase activity (*p* < 0.001) driven by the pGL4.23-6TCF (Wnt/β-catenin pathway) reporter vector following injection of rxLeptin. Forced expression of constitutively active β-catenin confirmed the activity of the pGL4.23-6TCF reporter (47-fold increase; *p* < 0.0001).

## Discussion

Leptin has well-established roles in adult physiology, but its developmental actions are less understood. Here, we show that *lep* expression increases strongly during tadpole metamorphosis in parallel with an increase in mitosis in tadpole brain. Also, i.c.v. injection of rxLeptin induced mitosis in neurogenic zones of premetamorphic tadpole brain. Gene expression screening identified genes regulated by leptin signaling in early premetamorphic tadpole brain, and highlighted several signaling pathways (Wnt/β-catenin, TGFβ, hedgehog, and insulin signaling) through which leptin may effect its regulatory role in neurogenesis. We also show that leptin activates Wnt/β-catenin pathway signaling in tadpole brain *in vivo*, as evidenced by activation of a Wnt/β-catenin pathway reporter vector.

### Leptin Promotes Neurogenesis in *Xenopus* Tadpole Brain

We observed a strong induction of mitosis in neurogenic zones of the premetamorphic tadpole brain following i.c.v. injection of rxLeptin. The rapid increase in pSTAT3-ir in cells of these regions 1 h after rxLeptin injection supports that they express functional LepR, and therefore that the action of leptin on mitosis is likely to be direct. In rodent embryonic/fetal brain, LepR mRNA is expressed within the VZ/SVZ of the 3V (18, 20). The LepR mRNA level in the VZ/SVZ declines through development, after which time it appears in the arcuate nucleus and ventromedial hypothalamus (18). These LepR mRNA-expressing NSCs may be precursors of hypothalamic feeding control and hypophysiotropic neurons of the adult (21). The LepR is also expressed in the VZ/SVZ (progenitor cell niches) of adult monkey brain (53).

Several lines of evidence from studies in rodents support that leptin controls cell proliferation in developing brain and can also induce neurogenesis in adults. For example, the reduced brain weight and DNA content in *ob/ob* mice is restored by leptin injection (4, 8), and leptin has been found to induce mitosis in rodent brain *in vivo* (8–10), and in tissue culture (11, 12). The large increase in *lep* mRNA in tadpoles during metamorphosis, a postembryonic developmental period that has been compared to the neonatal/early postnatal period in direct developing species like mammals (54), may be similar, both phenomenologically and functionally, to the postnatal leptin surge that occurs in rodents (17–19). It has been hypothesized that this postnatal increase in leptin plays a pivotal role in the development of the hypothalamic feeding control circuitry (13, 15, 16, 55–56). Leptin has also been shown to promote neurogenesis in the hippocampus of adult mammals (57), which may depend on Wnt/β-catenin signaling (58). The extra-hypothalamic actions of leptin on adult neurogenesis and neural plasticity may improve cognition and mood in animal models of depression and anxiety, and circulating leptin concentration is negatively correlated with Alzheimer’s disease in humans (59), and promotes neurogenesis in a mouse model of Alzheimer’s disease (60). Leptin has also been shown to be neuroprotective and induces neurogenesis and angiogenesis after stroke (61–63).

Cell proliferation in tadpole brain during metamorphosis depends on thyroid hormone (30). However, other mitogens such as leptin, activators of the TGFβ pathway, neurotrophins, and insulin-like peptides likely also play important roles in this developmental process. Indeed, there may be synergy between different signaling pathways, some that are affected by nutritional state like leptin and insulin-like peptides, that determine cell expansion and development of neural structures and pathways. Leptin has been found to synergize with thyroid hormone to induce proliferation of chondrocytes in growth plates, and also to promote terminal differentiation (64). Whether similar synergy occurs in the developing brain, which is critically dependent on thyroid hormone for its development, requires further study.

### Leptin Activates Canonical Wnt/β-Catenin Signaling in *Xenopus* Tadpole Brain

Using gene expression screening, we discovered sets of genes that are rapidly induced or repressed by leptin signaling in the tadpole preoptic area/hypothalamus. The major pathways regulated by leptin included the Wnt/β-catenin and TGFβ signaling pathways. Notably, we identified several genes involved in the Wnt/β-catenin signaling pathway, including β-catenin, the central component of the canonical Wnt/β-catenin pathway. The Wnt proteins, and the canonical Wnt/β-catenin intracellular signaling pathway, play central roles in development and disease (65), including development of the central nervous system, and are known to promote cell proliferation (66). The Wnt ligands bind to cell surface receptors of the Frizzled (Fz) and low-density lipoprotein-related protein families (67). In canonical Wnt/β-catenin signaling, the absence of Wnt ligand causes cytoplasmic signaling components such as glycogen synthase kinase 3β (GSK3β) to phosphorylate β-catenin, leading to the latter’s degradation. Activation of Wnt receptors leads to phosphorylation of GSK3β, allowing β-catenin to accumulate in the cytoplasm. The accumulated β-catenin translocates to the nucleus, where it interacts with lymphoid enhancer-binding factor 1 with lymphoid enhancer-binding factor 1 (also known as TCF) to activate transcription of Wnt/β-catenin target genes (67, 68).

In addition to the Wnt receptor Fzd10 and β-catenin (ctnnb1), one of the TCFs, Tcf7l1 (also known as TCF-3) was induced by leptin in tadpole brain. This transcription factor is a transcriptional repressor (67) and was recently found to promote growth of colorectal cancers (69). *Myc* was also identified in our screen and is a Wnt/β-catenin target gene that functions in mitosis (70). The cyclin-dependent kinase 10 was induced by leptin and is known to be modulated by Wnt/β-catenin signaling (66, 71).

There is mounting evidence from different systems and tissues that leptin induction of mitosis and cell survival depends on Wnt/β-catenin signaling (72, 73). For example, the level of β-catenin in the cytoplasm is maintained low through continuous proteasome-mediated degradation controlled by a protein complex of GSK3/APC/Axin (65). Phosphorylation of GSK3 on serine 9 by activated Wnt/β-catenin signaling leads to destruction of this complex and the accumulation of β-catenin. β-Catenin translocates to the nucleus to regulate gene transcription (65). Leptin acts as a growth factor for different kinds of tumor cells (73, 74), causes rapid nuclear translocation of β-catenin, and activates other components of the Wnt/β-catenin pathway (74–76). Leptin-dependent activation of Wnt/β-catenin signaling has also been shown to play a role in leptin action in the adult hypothalamus to modulate glucose homeostasis and energy balance (77–80).

### Leptin Modulates TGFβ, Hedgehog, and Insulin Signaling in *Xenopus* Tadpole Brain

After the Wnt/β-catenin pathway, the next most enriched pathway activated by leptin in tadpole brain was that regulated by the TGFβ superfamily of ligands, in particular bone BMP and activin. The TGFβ signaling pathway plays central roles in animal development, including cell proliferation, cell differentiation, and apoptosis (81, 82). Several components of this pathway were modulated by leptin in tadpole brain, including ligands, cell surface receptors, and receptor-activated transcription factors of the SMAD family (see Figure 6).

The hedgehog signaling pathway was also found to be activated by leptin (Figure S1 in Supplementary Material). A proposed mechanism of leptin-induced neurogenesis in murine transient amplifying neuroblasts involves hedgehog signaling regulation (83). We also found that leptin modulated components of the insulin signaling pathway (see Figure S2 in Supplementary Material). Interactions between leptin and insulin signaling, and potential therapeutic uses for leptin in normalizing type 2 diabetes have been described recently (84–86). The signal transduction pathways initiated by insulin and leptin are both distinct and overlapping. For example, leptin activates the JAK2–STAT pathway, while insulin activates the mitogen-activated protein kinase pathway. Both insulin and leptin appear to activate the PI3K pathway. Recent findings show that insulin can potentiate leptin signaling (87).

### Leptin and Developmental Programming

Early life nutrition (from maternal source—placenta or yolk, or from feeding—larva) can influence adipocyte production of leptin in the fetus (88, 89) or larva (*Xenopus*, Melissa Cui Bender and Robert J. Denver, unpublished data). Alterations in hormone production, influenced by nutrition during critical periods of development can affect the timing of development, and exert lasting effects on the structure and function of hypothalamic feeding and neuroendocrine circuits, a phenomenon termed developmental programming (88–93). There is mounting evidence that early life nutrition is an important determinant for risk of obesity (94), and modulation of leptin signaling during critical developmental periods can have long-term consequences for adult physiology (95–99). In animal models, undernutrition during pregnancy leads to a premature leptin surge, and offspring develop leptin resistance as adults, especially following a high-fat diet (100). A decrease or loss of leptin signaling in early postnatal life impairs development of the feeding circuit, and also predisposes individuals to leptin resistance and obesity as adults (95–99, 101–103).

The cellular and molecular mechanisms by which leptin acts on the developing brain to “program” the hypothalamic feeding control circuit are poorly understood (104). One possible mechanism of action is for circulating leptin in the neonate/tadpole to act within neurogenic zones of the developing brain to induce expansion of a LepR expressing cell population, which ultimately establishes the leptin-responsive network in the hypothalamus and other brain regions. Mammals develop competence to respond to leptin signaling during early postnatal development (15, 16, 105); amphibians develop competence to respond to leptin during early premetamorphosis (Melissa Cui Bender and Robert J. Denver, unpublished data). Functional LepR is detected in the VZ/SVZ during early postnatal development in rodents (18) and premetamorphosis in *Xenopus* (Figure 2D). Based on our findings and those in the literature, we hypothesize that leptin acts on the neural progenitor population *via* Wnt/β-catenin signaling to induce mitosis and promote cell survival.

Future fate mapping studies can investigate if cells born in the ependymal layer following leptin injection migrate to and populate the preoptic area and ventral hypothalamus. It will also be interesting to investigate the consequences of aberrant leptin signaling during early postembryonic development on later-life physiology and hypothalamic feeding control centers using the *Xenopus* model system. Leptin signaling in early stage *Xenopus* tadpole brain can be easily activated by i.c.v. injection of rxLeptin. Furthermore, cells in the tadpole VZ/SVZ can be transfected by EM gene transfer with expression plasmids (e.g., pCS2-xLepR) to force LepR expression in stem/progenitor cells, followed by injection of rxLeptin i.c.v. to activate LepR signaling. We predict that forced expression of LepR in progenitor/stem cells in the developing brain will promote proliferation followed by differentiation of these cells into the LepR expressing lineage.

## Ethics Statement

All procedures involving animals were conducted under an approved animal use protocol (PRO00006809) in accordance with the guidelines of the Institutional Animal Care and Use Committee at the University of Michigan.

## Author Contributions

MB participated in the design of experiments, conducted the leptin mRNA analysis, immunohistochemistry for pH3, DNA microarray analyses and electroporation-mediated gene transfer, and participated in writing the manuscript. CS did the bioinformatics analyses of the DNA microarray data and participated in writing the manuscript. RD participated in the design of experiments, analysis of the data, and in writing the manuscript.

## Conflict of Interest Statement

The authors declare that the research was conducted in the absence of any commercial or financial relationships that could be construed as a potential conflict of interest. The handling editor declared a shared affiliation, though no other collaboration, with the authors, and the handling editor states that the process met the standards of a fair and objective review.
