# Supplementary figures and images for: Leptin Induces Mitosis and Activates the Canonical Wnt/β-Catenin Signaling Pathway in Neurogenic Regions of Xenopus Tadpole Brain
Source: Front Endocrinol (Lausanne). 2017 May 8;8:99. doi: 10.3389/fendo.2017.00099 (PMC5421298; doi:10.3389/fendo.2017.00099)

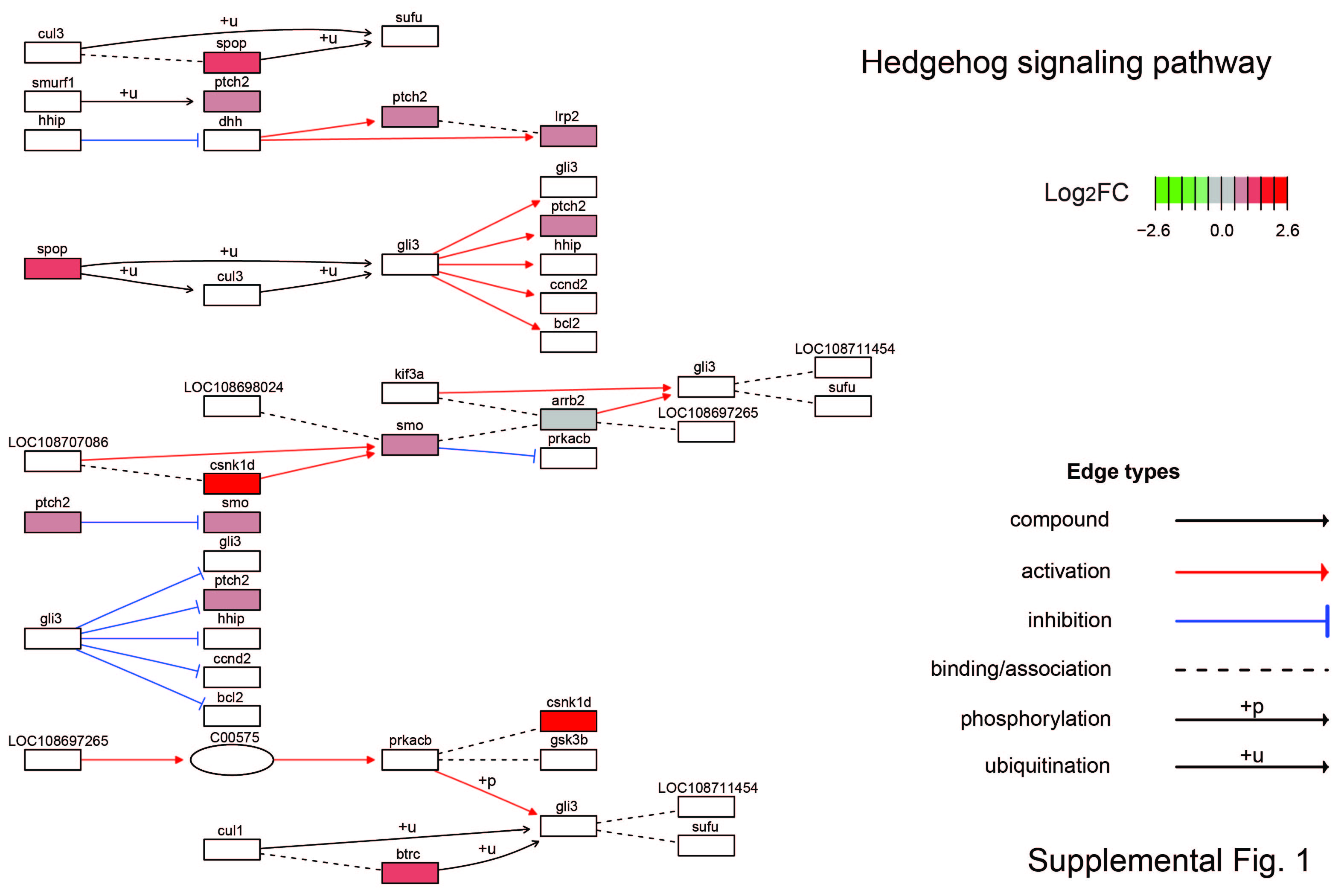

Supplement: Supplementary file 4 [file Image_1.JPEG]

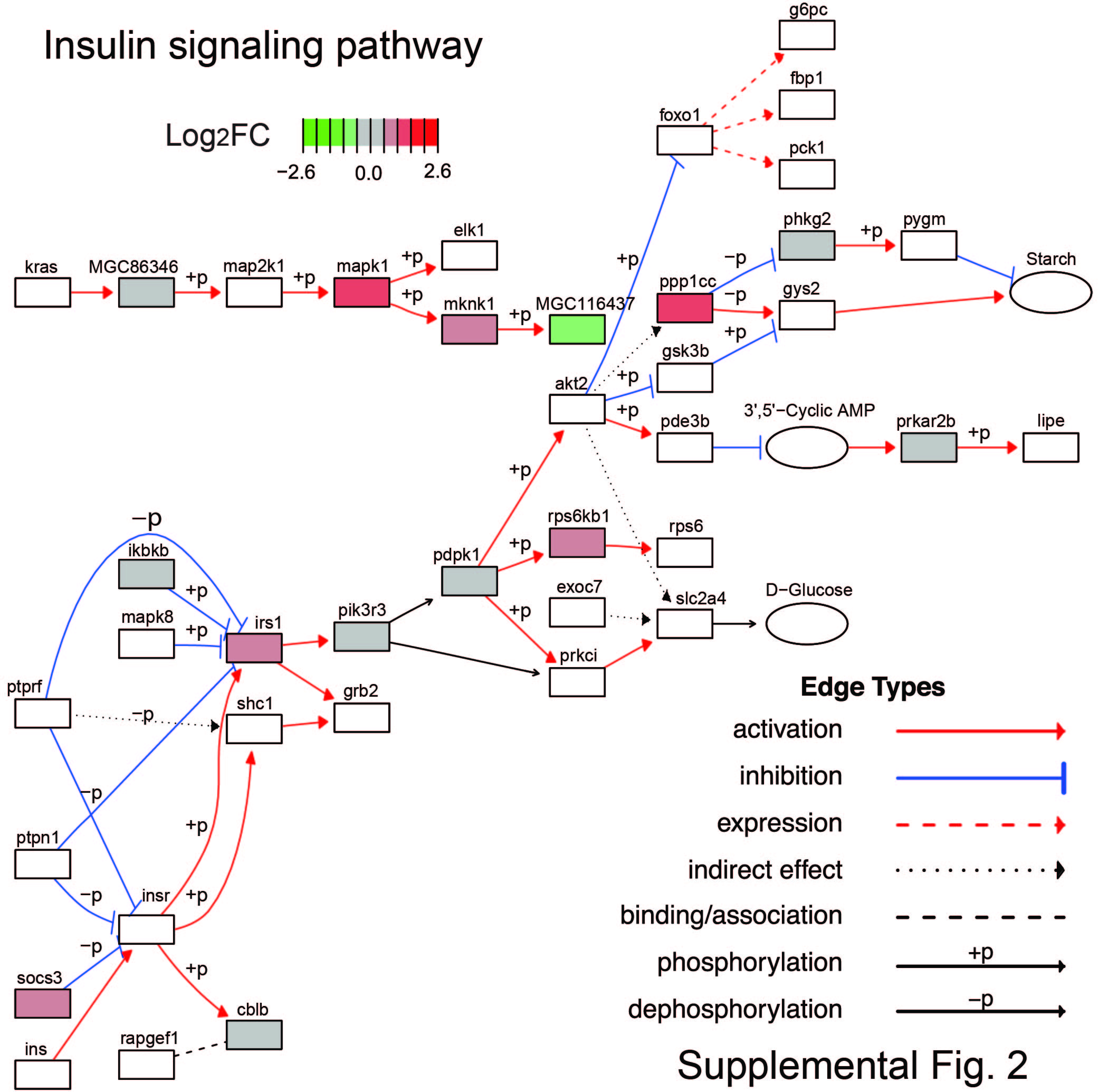

Supplement: Supplementary file 5 [file Image_2.JPEG]
